# Contemporary Treatment Patterns and Oncological Outcomes of Metastatic Hormone-sensitive Prostate Cancer and First- to Sixth- line Metastatic Castration-resistant Prostate Cancer Patients

**DOI:** 10.1016/j.euros.2024.06.010

**Published:** 2024-06-27

**Authors:** Mike Wenzel, Carolin Siech, Benedikt Hoeh, Florestan Koll, Clara Humke, Derya Tilki, Thomas Steuber, Markus Graefen, Séverine Banek, Luis A. Kluth, Felix K.H. Chun, Philipp Mandel

**Affiliations:** a Department of Urology, University Hospital Frankfurt, Goethe University Frankfurt am Main, Frankfurt, Germany; b Martini-Klinik Prostate Cancer Center, University Hospital Hamburg-Eppendorf, Hamburg, Germany; c Department of Urology, University Hospital Hamburg-Eppendorf, Hamburg, Germany; d Department of Urology, Koc University Hospital, Istanbul, Turkey

**Keywords:** Metastatic prostate cancer, Metastatic hormone-sensitive prostate cancer, Metastatic castration-resistant prostate cancer, Castration refractory, De novo, Recurrent

## Abstract

**Background and objective:**

With approval of novel systemic therapies within the past decade for metastatic hormone-sensitive (mHSPC) and castration-resistant (mCRPC) prostate cancer, patients may receive several therapy lines. However, the use of these treatments is under an ongoing change. We investigated contemporary treatment trends and progression-free (PFS) and overall (OS) survival of different therapy lines.

**Methods:**

Relying on our institutional tertiary-care database, we identified mHSPC and mCRPC patients. The main outcome consisted of treatment changes (estimated annual percentage change [EAPC]) within the past decade, as well as PFS and OS for different mHSPC and mCRPC treatment lines.

**Key findings and limitations:**

In 1098 metastatic patients, the median age was 70 yr with a median of two systemic therapy lines. For first-line mCRPC between 2013 and 2023, androgen deprivation monotherapy (ADT) monotherapy usage decreased significantly from 31% to 0% (EAPC −38.3%, *p* < 0.001), while the administration of chemotherapy increased from 16.7% to 33.3% (EAPC: +10.1%, *p* < 0.001). The PFS/OS rates of mHSPC patients was 21/67 mo, and those for first-, second-, third-, fourth-, fifth-, and sixth-line mCRPC patients were 11/47, eight of 30, seven of 24, six of 19, seven of 17, and seven of 13 mo, respectively. With an increased number of new combination therapy lines received, the median OS in mCRPC improved from 26 mo (one systemic treatment) to 52 mo (two or more lines of systemic treatment).

**Conclusions and clinical implications:**

Significant changes in treatment patterns could be observed for mHSPC and mCRPC patients within the past decade, and usage of ADT monotherapy has decreased rapidly in real-world practice. Moreover, PFS decreases significantly with every therapy line, and OS increases with the implementation of new therapies.

**Patient summary:**

Improvements in the real-world setting regarding the usage of combination therapies for metastatic hormone-sensitive and castration-resistant prostate cancer were made, which is reflected in contemporary survival outcomes.

## Take Home Message

Intensified combination therapies have been adopted in the real-world setting of metastatic hormone-sensitive prostate cancer and metastatic castration-resistant prostate cancer (mCRPC) patients within the past decade, and treatment trends follow guidelines and phase 3 derived recommendations/results. Contemporary oncological outcomes such as progression-free and overall survival in advanced mCRPC treatment lines still reveal appropriate outcomes.

## 1 Introduction

Within the past two decades, management of metastatic hormone-sensitive (mHSPC) and castration-resistant (mCRPC) prostate cancer changed significantly from androgen deprivation monotherapy (ADT) toward intensified combination therapies, and is still an area of extensive ongoing uro-oncological research [1], [2], [3], [4], [5], [6], [7], [8].

With increasing treatment options for either mHSPC or mCRPC stage, patients will receive multiple treatment sequences during their cancer care [9]. Moreover, it seems that differences in treatment sequences may also affect oncological outcomes [10]. Furthermore, even after receiving multiple therapy lines, mCRPC patients harbor acceptable life expectancy, which might decrease after every therapy line [11], [12]. However, with the approval of novel treatment options, increasing life expectancy in general, and adoption and improvement of health care systems, trends in systemic mCRPC treatments may differ significantly between more historical and more contemporarily treated mCRPC patients. Moreover, adoption of recently approved therapeutic agents for mHSPC and mCRPC may also significantly influence progression-free survival (PFS) as well as overall survival (OS) of the patients. For example, recent approval of triplet therapies for mCRPC patients, a combination of ADT plus androgen receptor signaling inhibitor (ARSI) and poly-(ADP-ribose)-polymerase inhibitor (PARPi), has shown promising synergistic effects [13]. However, currently data on real-world outcomes of mHSPC and mCRPC treatment trends within the recent decade are scant. Moreover, PFS and OS outcomes often mainly focus on mHSPC and first and second treatment lines of mCRPC [14].

We addressed this void and relied on our institutional metastatic prostate cancer database to identify treatment trends and cancer-control outcomes of contemporarily treated mHSPC and mCRPC patients. We hypothesized that significant and clinically meaningful differences in mHSPC and mCRPC treatment patterns may be uncovered. Moreover, we hypothesized that, with adoption of intensified combination therapies for mHSPC and mCRPC, PFS and OS outcomes in all therapy lines may exceed those of more historical reports.

## 2 Patients and methods

### 2.1 Study population

After the approval of the local ethics committee of the Goethe University Hospital Frankfurt (reference number: SUG-5-2018) and in accordance with the principles of the Declaration of Helsinki, we retrospectively identified all mHSPC and subsequent castration-resistant prostate cancer patients treated at the Department of Urology, University Hospital Frankfurt, Germany (*n* = 1098). The inclusion period consisted of all metastatic prostate cancer patients discussed in an interdisciplinary tumor conference since 2014. Patients with unknown follow-up status were excluded from the current study. These criteria yielded 1098 mHSPC and subsequently 674 mCRPC patients.

### 2.2 Statistical analysis and study endpoints

Descriptive statistics included the frequencies and proportions of categorically coded variables utilized in the analysis, while median values and interquartile ranges (IQRs) were presented for continuously coded variables. Statistical significance in proportion differences was evaluated using the chi-square test, while differences in distributions were analyzed using the *t* test and Kruskal-Wallis test. Stratification of the patient cohort was performed according to time of metastatic onset (≤2017 vs 2017–2024) to explore the most recent trends and outcomes.

For OS and PFS analyses, Kaplan-Meier estimates were used and depicted graphically. Seven separate sets of analyses were computed for mHSPC toward sixth-line mCRPC patients. Castration resistance and progression were defined as recently described, in accordance with the European Association of Urology criteria [15].

Sequential therapies of mHSPC toward first-, second-, and third-line mCRPC were depicted graphically by Sankey plots. Finally, the estimated annual percentage changes (EAPCs) in treatments of mCRPC patients over the past 5 and 10 yr were calculated as an exemplary presentation, using a log-linear regression model, of a recently described methodology [16]. All tests were two sided, with a level of significance set at *p* < 0.05, and R software environment for statistical computing and graphics (version 2023.12.1+402; R Foundation for Statistical Computing, Vienna, Austria) was used for all analyses.

## 3 Results

Overall, 1098 metastatic prostate cancer patients could be included for a further analysis with a median follow-up of 32 mo (IQR: 11–60 mo). The median age at prostate cancer diagnosis was 66 yr (IQR 61–72 yr) and the median age at metastatic prostate cancer diagnosis was 70 yr (IQR: 64–76 yr), with a prostate-specific antigen (PSA) value of 43 ng/ml (IQR: 11–223 ng/ml; Table 1). Proportions of Eastern Cooperative Oncology Group ≥2 were 6.5%.

**Table 1:** Characteristics of 1098 metastatic prostate cancer (mPCa) patients stratified according to the year of metastatic onset

Characteristic	*N*	Overall (*N* = 1098)^a^	Years ≤2006–2017 (*N* = 528, 48%)^a^	Years 2018–2024 (*N* = 570, 52%)^a^	*p* value
Follow-up (mo)	802	32 (11, 58)	54 (28, 87)	20 (5, 41)	<0.001
Age at PCa diagnosis (yr)	1096	66 (61, 72)	66 (59, 71)	67 (61, 73)	0.004
Age at mPCa diagnosis (yr)	1029	70 (64, 76)	69 (63, 74)	71 (64, 77)	<0.001
PSA at mHSPC (ng/ml)	561	43 (11, 223)	57 (12, 403)	40 (11, 175)	0.10
PSA nadir at mHSPC (ng/ml)	378	0.5 (0.07, 2.9)	1.1 (0.3, 4.3)	0.3 (0.05, 2.0)	<0.001
PSA decline ≥99%	316	153 (48)	31 (39)	122 (52)	0.045
PSA at mCRPC (ng/ml)	374	15 (4, 65)	18 (5, 65)	13 (4, 64)	0.2
PSA at 2nd-line mCRPC (ng/ml)	321	46 (11, 137)	46 (12, 122)	44 (9, 140)	0.7
PSA at 3rd-line mCRPC (ng/ml)	236	66 (19, 214)	66 (22, 186)	73 (19, 391)	0.5
PSA at 4th-line mCRPC (ng/ml)	169	114 (40, 299)	118 (41, 273)	112 (35, 314)	0.8
PSA at 5th-line mCRPC (ng/ml)	89	204 (63, 493)	219 (63, 493)	157 (65, 507)	0.9
PSA at 6th-line mCRPC (ng/ml)	44	238 (80, 795)	215 (72, 512)	635 (156, 1,588)	0.11
Treatment sequences mCRPC	1089	2.00 (2.00, 4.00)	3.00 (2.00, 5.00)	2.00 (2.00, 3.00)	<0.001
ECOG ≥2	796	52 (6.5)	35 (9.1)	17 (4.1)	<0.001
Gleason score 8–10	964	656 (68)	310 (70)	346 (66)	0.14
cT stage	447	168 (38)	36 (30)	132 (40)	0.058
Local therapy RP/RT	1098	439 (40)	228 (43)	211 (37)	0.037
pT3–4 after RP	373	276 (74)	147 (74)	129 (74)	>0.9
pN1 after RP	525	228 (44)	81 (40)	147 (46)	0.2
Visceral metastasis at mPCa	92	75 (8.3)	29 (7.7)	46 (8.8)	0.6
High-volume mHSPC	655	327 (50)	121 (57)	206 (46)	0.01
High-risk mHSPC	670	364 (54)	135 (62)	229 (51)	0.01
De novo mHSPC	1076	645 (60)	298 (58)	347 (62)	0.2
Systemic therapy mHSPC	1054				<0.001
ADT mono nmHSPC/nmCRPC		602 (57)	407 (80)	195 (36)	
Docetaxel		105 (10.0)	47 (9.2)	58 (11)	
ARSI		302 (29)	44 (8.6)	258 (47)	
Triplet		25 (2.4)	0 (0)	25 (4.6)	
Other		20 (1.9)	12 (2.4)	8 (1.5)	
Systemic therapy mCRPC	1098				<0.001
ADT mono		70 (6.4)	65 (12)	5 (0.9)	
Chemotherapy		132 (12)	79 (15)	53 (9.3)	
Lu-RLT		24 (2.2)	10 (1.9)	14 (2.5)	
ARSI		364 (33)	218 (41)	146 (26)	
Radium		32 (2.9)	31 (5.9)	1 (0.2)	
Systemic therapy 2nd-line mCRPC	1098				<0.001
Chemotherapy		158 (14)	102 (19)	56 (9.8)	
Lu-RLT		65 (5.9)	31 (5.9)	34 (6.0)	
ARSI		224 (20)	173 (33)	51 (8.9)	
PARPi ± ARSI		7 (0.6)	3 (0.6)	4 (0.7)	
Radium		28 (2.6)	28 (5.3)	0 (0)	
Systemic therapy 3rd-line mCRPC	1098				<0.001
Chemotherapy		103 (9.4)	84 (16)	19 (3.3)	
Lu-RLT		99 (9.0)	62 (12)	37 (6.5)	
ARSI		110 (10)	81 (15)	29 (5.1)	
PARPi ± ARSI		6 (0.5)	1 (0.2)	5 (0.9)	
Radium		19 (1.7)	19 (3.6)	0 (0)	

Data are presented as *n* (%).

ADT mono = androgen deprivation monotherapy; ARSI = androgen receptor signaling inhibitor; ECOG = Eastern Cooperative Oncology Group; mCRPC = metastatic castration-resistant prostate cancer; Lu-RLT = lutetium radioligand therapy; mHSPC = metastatic hormone-sensitive prostate cancer; nmHSPC/nmCRPC = nonmetastatic hormone-sensitive/castration-resistant prostate cancer; PARPi = poly-(ADP-ribose)-polymerase inhibitor; PCa = prostate cancer; PSA = prostate-specific antigen; RP = radical prostatectomy; RT = radiotherapy.

In median, all included patients received two treatment lines (IQR: 2–4) and were diagnosed predominately with metastatic disease within recent years (2018–2024; 52%). Overall, 68% of included patients harbored Gleason scores 8–10 and 40% received a local therapy with radical prostatectomy or radiation therapy. Of the patients with radical prostatectomy, 74.0% and 40% harbored pT3–4 and pN1 stages in final pathology. Moreover, 60% were de novo metastatic prostate cancer patients. At diagnosis of mHSPC, 50% and 54% were classified as high volume according to the CHAARTED criteria and high risk according to the LATITUDE criteria. Visceral metastases were present in 8.3% at the occurrence of metastatic disease.

### 3.1 Treatment patterns, sequences, and trends in mHSPC and mCRPC

**Fig. 1:**
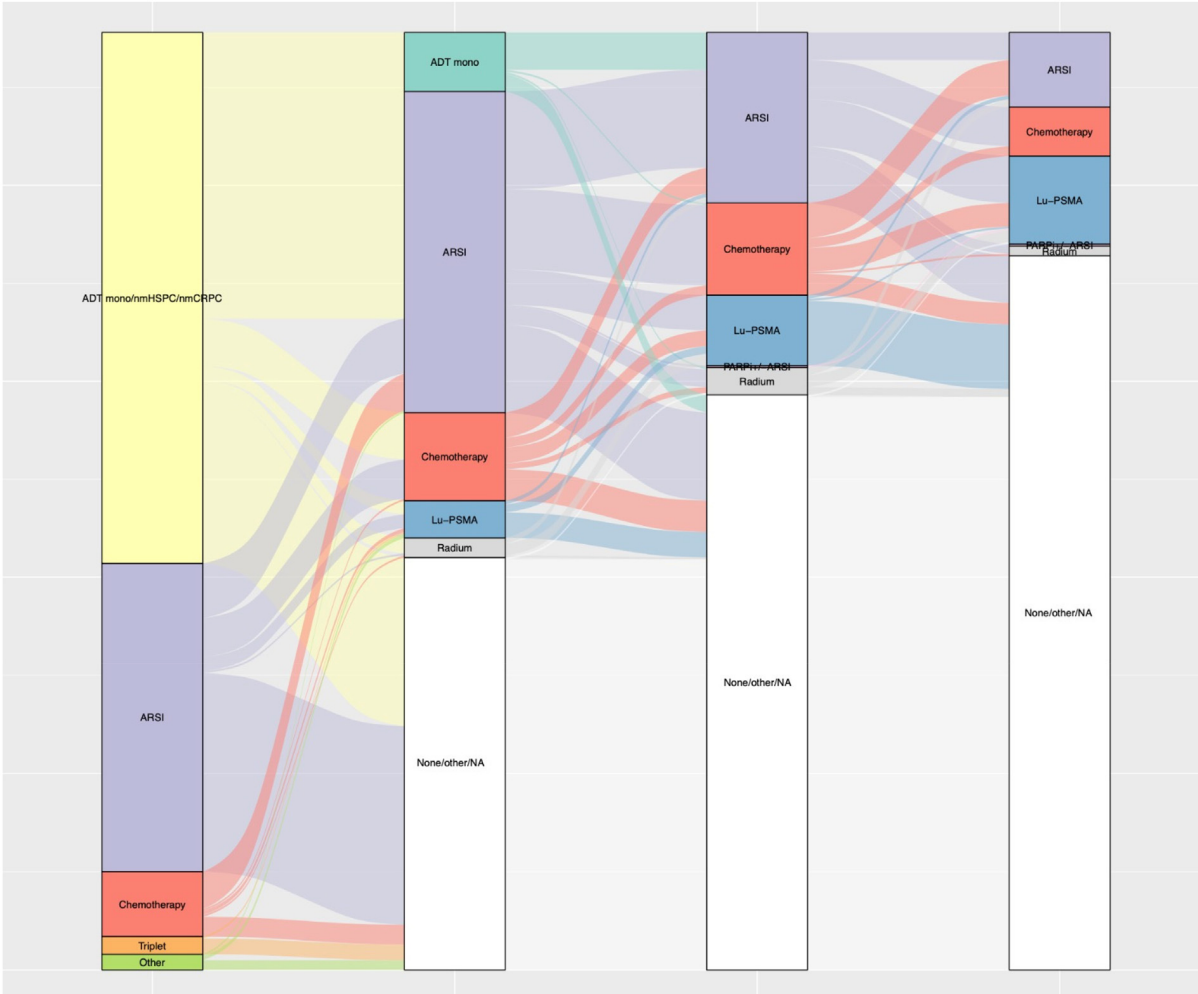
Sankey plot depicting treatment sequences between metastatic hormone-sensitive prostate cancer (mHSPC) and first- and second-line metastatic castration-resistant prostate cancer patients (mCRPC). ADT mono = androgen deprivation monotherapy; ARSI = androgen receptor signaling inhibitor; Lu-PSMA = lutetium prostate-specific membrane antigen therapy; NA = not available; nmHSPC/nmCRPC = nonmetastatic hormone-sensitive/castration-resistant prostate cancer; PARPi = poly(ADP-ribose)-polymerase inhibitor.

Detailed treatment distribution for mHSPC and first-, second-, and third-line mCRPC is displayed in Table 1 and stratified according to the year of metastatic onset. Moreover, treatment sequences are additionally displayed in a Sankey plot (Fig. 1). The median PSA increased from 15 ng/ml in first-line mCRPC (IQR 4–65 ng/ml) to 223 ng/ml in sixth-line mCRPC (IQR: 80–795 ng/ml).

**Fig. 2:**
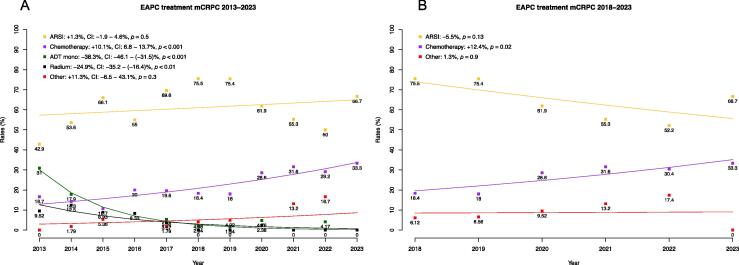
Estimated annual percentage changes (EAPCs) in treatment patterns of metastatic castration-resistant prostate cancer (mCRPC) patients between (A) 2013–2023 and (B) 2018–2023. ADT mono = androgen deprivation monotherapy; ARSI = androgen receptor signaling inhibitor; CI = confidence interval.

The proportions of treatment patterns of mHSPC, and first-, second-, and third-line mCRPC patients differed significantly after stratification regarding the year of metastatic onset, with more intensified combination therapies in the recent years. To depict treatment trends toward more intensified therapies within the recent years more clearly, we performed an exemplary presentation of EAPC analyses for first-line mCRPC patients. Within the past 10 yr of observation period (2013–2023; Fig. 2A), the administration of ADT monotherapy decreased significantly from 31% in 2013 to 0% in 2023, with an EAPC of –38.3% (*p* < 0.001). Similarly, the administration of radium-223 also decreased significantly, with treatment rates of 9.5% in 2013 and 0% in 2023 (EAPC: –24.9%, *p* < 0.001). Conversely, administration of chemotherapy increased from 16.7% in 2013 to 33.3% in 2023 (EAPC: +10.1%, *p* < 0.001). Moreover, the administration of ARSIs also increased from 42.9% to 66.7% (EAPC: +1.3%, *p* = 0.5). A similar trend toward more intensified therapies can be seen in mHSPC and all mCRPC therapy lines.

Looking more specifically into the past 5 yr of treatment (Fig. 2B), a decrease of the usage of ARSIs in favor of chemotherapy could be observed.

### 3.2 PFS and OS in mHSPC patients

**Fig. 3:**
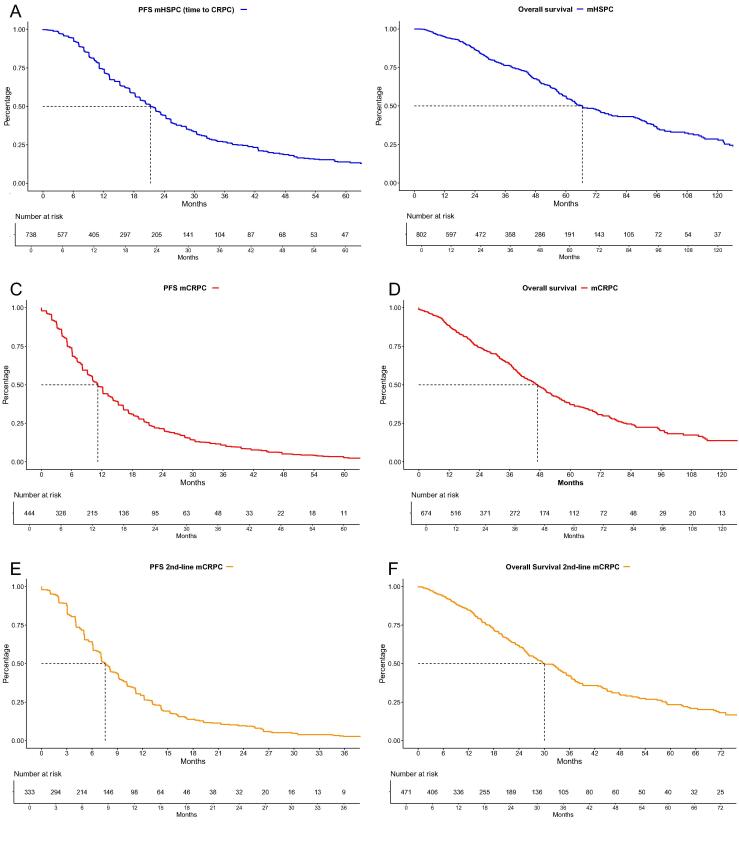
Progression-fee (PFS) and overall (OS) survival of (A and B) metastatic hormone-sensitive prostate cancer (mHSPC) patients, and (C and D) first-line and (E and F) second-line metastatic castration-resistant prostate cancer (mCRPC) patients. CRPC = castration-resistant prostate cancer.

In Kaplan-Meier estimates of all included mHSPC patients, the time to mCRPC (PFS) was 21 mo (95% confidence interval [CI]: 19–23 mo; Fig. 3A), with 1- and 2-yr PFS rates of 74% and 44%, respectively. Regarding OS analyses, the median OS was calculated to be 67 mo (95% CI: 62–77 mo; Fig. 3B), with 3-, 5-, and 8-yr OS rates of 76%, 56%, and 36%, respectively.

### 3.3 PFS and OS in first- and second-line mCRPC patients

After progression to mCRPC, the median time to further progression was 11 mo (95% CI: 10–12 mo; Fig. 3C), with 1- and 2-yr PFS rates of 48% and 22%, respectively. The median OS in first-line mCRPC was 47 mo (95% CI: 42–51 mo; Fig. 3D), with 3-, 5-, and 8-yr OS rates of 64%, 37%, and 20%, respectively.

In second-line mCRPC patients, the median PFS was 8 mo (95% CI: 7–9 mo; Fig. 3E), with 1- and 2-yr PFS rates of 29% and 9.6%, respectively. Moreover, the median OS in second-line mCRPC patients was 30 mo (95% CI: 26–34 mo; Fig. 3F) with 3-, 5-, and 8-yr OS rates of 41%, 23%, and 12%, respectively.

Owing to adherence to increased administration of systemic therapy lines in mCRPC, we additionally performed analyses of patients receiving different numbers of life-prolonging treatments. By having an increased number of new systemic (combination) therapy lines at hand, median OS improved further, for example, from 26 mo (one systemic treatment) to 52 mo (two or more lines of systemic treatment).

### 3.4 PFS and OS in advanced lines of mCRPC patients

**Fig. 4:**
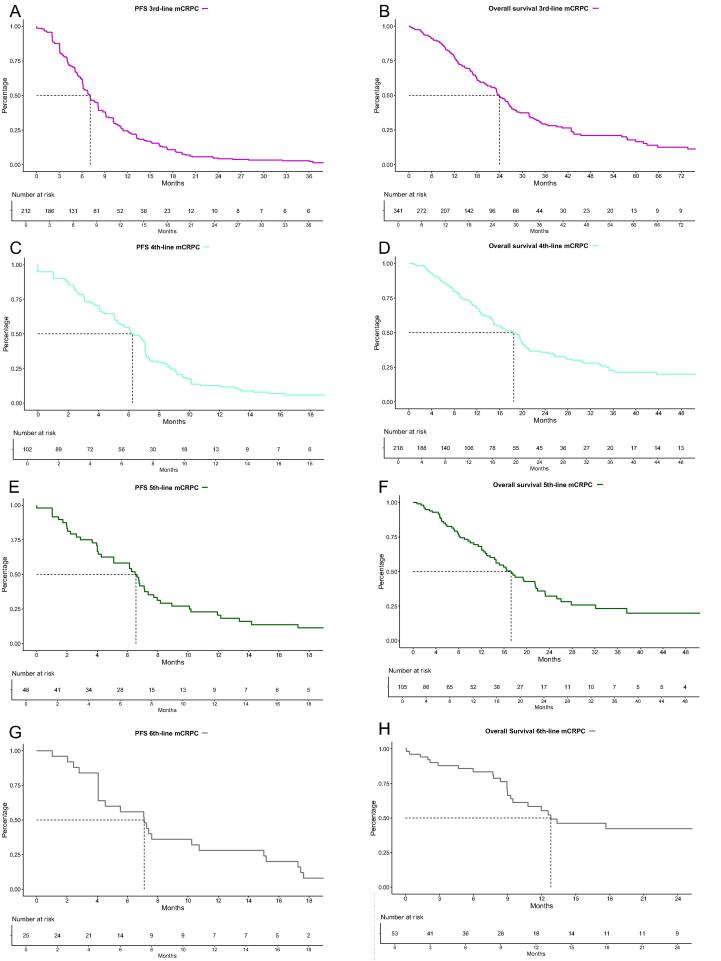
Progression-fee (PFS) and overall (OS) survival of (A and B) third-line, (C and D) fourth-line, (E and F) fifth-line, and (G and H) sixth-line metastatic castration-resistant prostate cancer (mCRPC) patients.

In third-line mCRPC patients, the median PFS was 7 mo (95% CI: 6–8 mo; Fig. 4A), with 6-, 12-, and 18-mo PFS rates of 62%, 25%, and 11%, respectively. In OS analyses of third-line mCRPC patients, the median OS was 24 mo (95% CI: 22–27 mo; Fig. 4B), with 1-, 2-, and 3-yr OS rates of 78%, 49%, and 39%, respectively.

In fourth-, fifth-, and sixth-line mCRPC patients, the median PFS rates were 6 mo (95% CI: 5–7 mo; Fig. 4C), 7 mo (95% CI: 5–8 mo; Fig. 4E), and 7 mo (95% CI: 4–15 mo; Fig. 4G), respectively. The median OS of fourth-, fifth-, and sixth-line mCRPC patients were 19 mo (95% CI: 15–20 mo; Fig. 4D), 17 mo (95% CI: 14–22 mo; Fig. 4F), and 13 mo (95% CI: 9–35 mo; Fig. 4H). Corresponding 6-, 12-, and 24-mo OS rates were 87%, 68%, and 36% for fourth-line mCRPC; 83%, 68%, and 32% for fifth-line mCRPC, and 83%, 55%, and 42% for sixth-line mCRPC patients, respectively.

## 4 Discussion

We hypothesized that significant and clinically meaningful differences in mHSPC and mCRPC treatment patterns may be unraveled within the recent years with the adoption of intensified combination therapies showing improvement of OS. Moreover, we hypothesized that PFS and OS outcomes in mHSPC and in therapy lines for mCRPC may exceed those of more historical reports, and that PFS decreases with every further line of therapy line. We tested these hypotheses within our institutional metastatic prostate cancer database and made several important observations.

First, we observed noteworthy baseline and tumor characteristics of all included 1098 metastatic prostate cancer patients. Specifically, the median age at metastatic disease was 70 yr, the majority harbored pT3–4 at final pathological report after local therapy, and the rate of de novo metastatic disease was 60%. Moreover, 50% and 54% were classified as high volume according to the CHAARTED criteria and high risk according to the LATITUDE criteria, respectively [17], [18]. Comparing our real-world observations with the data derived from phase 3 trials, some differences in tumor characteristics can be observed. For example, in the TITAN trial, the median age at metastatic disease was comparable (69/68 yr), while the rates of de novo mHSPC (78%/84%) or high-volume disease (62%/64%) were higher in the apalutamide than in the placebo arm [19]. However, comparing our data with previous real-world mHSPC data, a recent study by Kafka et al [20] also reported a rate of de novo mHSPC of 57%, with 50% high-volume and 52.5% high-risk characteristics. In consequence, the characteristics of metastatic prostate cancer patients in the real-world setting differ from those of study-selected metastatic prostate cancer patients. Moreover, it is of note that the rates of local therapies have decreased within our study cohort within the last years. One hypothesis may be the level 1 evidence derived from the STAMPEDE trial in 2018 showing an OS benefit in selected low-volume mHSPC patients only [21].

Second, we observed that treatment patterns in mHSPC and mCRPC patients differed significantly within the recent decade. More specifically as an exemplary presentation, we observed that intensified combination therapies were adopted in clinical practice, reducing the administration of ADT monotherapy to 0% in 2023 for first-line mCRPC patients, similar to the administration of radium-223. Conversely, the administration of docetaxel chemotherapy as first-line mCRPC treatment increased significantly, while ARSI usage was stable as the most administered treatment, which may be explained by more intensified administration of ARSIs in the mHSPC setting.

Moreover, the median number of systematic therapy lines administered for mCRPC was only 1 (IQR 1–3). These observations are in line with previous reported real-world data. For example, previous reports also demonstrated that an ARSI is the most frequently used systemic treatment in first-line mCRPC, and most patients receive only one treatment line for mCRPC [22], [23], [24], [25]. Moreover, the significant increase in the usage of docetaxel chemotherapy in our cohort may be explained by the effect of intensified administration of ARSIs as first-line treatment for mHSPC patients and a change of action within the subsequent mCRPC therapy, since an ARSI after ARSI treatment may lead to short PFS [10]. However, with the recent approval of triplet therapies for the mHSPC setting as a combination of ARSIs and docetaxel chemotherapy and its wide adoption in patients with a high metastatic burden, first-line mCRPC treatment may undergo another substantial change in its treatment patterns within the upcoming years. However, with the adoption of intensified therapies and multiple new systemic (combination) treatments for mCRPC patients, we additionally explored prolonged OS in patients receiving more than one systemic treatment. This is of note since it emphasizes the need of treatment change and the so-called “change of action” for life-prolonging systemic treatments [10].

Third, we observed that the median PFS and OS were, respectively, 21 and 68 mo for all mHSPC patients included. These observations are in line with the previously published reports. For example, in the CHAARTED trial, comparing intensified combination of ADT plus docetaxel, the median time to progression was 19 mo [17]. However, data from phase 3 trials of ARSI combination therapies exceed those of our overall cohort with several combination therapies [19], [26]. However, our data combine more historical patients receiving ADT monotherapy as well as patients with ARSI or triplet therapies, and therefore may explain those differences in real-world observations. Nonetheless, after stratification according to the year of metastatic onset, an increase in OS and PFS was observed within our study cohort.

Moreover, we observed that the median times of PFS and OS in first-line mCRPC patients were 11 and 47 mo, respectively. These observations exceed those made by previous, more historical patient cohorts. For example, in a study published by Bjartell et al [14], involving over 700 mCRPC patients treated within 2013–2018, the median PFS ranged from 6 to 9 mo and the median OS ranged from 22 to 32 mo, depending on the systemic treatment administered. Similar observations were made in a study of mCRPC patients from a Hong Kong patient cohort who were treated solely with enzalutamide (study period 2015–2017) [27]. This might additionally show that the introduction of new (combination) therapies in mCRPC also leads to longer OS. Comparing the PFS and OS data of more advanced mCRPC treatment lines, a recent multicenter study of cabazitaxel in combined fourth-, fifth-, and sixth-line mCRPC patients observed median PFS and OS of, respectively, 3 and 15 mo [12]. These data are comparable with our results, in which the median OS ranged from 19 mo (fourth-line mCRPC) to 13 mo (sixth-line mCRPC). It is especially of note that median PFS decreased with every additional treatment sequence. This observation indicates that clinicians should be aware of the more pronounced oncological treatment responses within the first treatment lines, and efforts should be made to provide best oncological treatments at the first metastatic diagnosis if patients are eligible. However, even after progression to advanced therapy lines, OS and PFS data seem still favorable compared with other urological tumors [28], [29].

Given the retrospective single-center design of our study, it is important to recognize its limitations. First, a potential selection bias inherent in retrospective analyses, as well as the specific characteristics of our patient cohort, may influence the results and limit their generalizability to broader populations and geographical regions, for example, histological subtypes [30]. Additionally, reliance on medical records raises concerns about potential incompleteness or inaccuracies, which could affect the reliability and validity of our findings. Moreover, the observational nature of the study and limitations in subgroup sample sizes such as PFS and OS analyses of advanced mCRPC patients or proportions of treatment sequences should also be considered. Therefore, although our study offers valuable insights, caution is advised when interpreting the findings and applying these to different contexts or populations. Finally, some of our ADT monotherapy patients may be distinguishable according to mHSPC/non-mHSPC or non-mCRPC status.

## 5 Conclusions

Taken together, our study implies intensified combination therapies have been adopted in the real-world setting of mHSPC and mCRPC patients within the last decade, and treatment trends follow guidelines and phase 3 derived recommendations/results. Moreover, we observed that contemporary oncological outcomes such as PFS and OS in advanced mCRPC treatment lines still reveal appropriate outcomes. However, efforts should be made that more mCRPC patients receive more than one systemic therapy line.

  ***Author contributions:*** Mike Wenzel had full access to all the data in the study and takes responsibility for the integrity of the data and the accuracy of the data analysis.

  *Study concept and design*: Wenzel, Siech, Mandel.

*Acquisition of data*: Wenzel, Siech, Hoeh.

*Analysis and interpretation of data*: Wenzel, Hoeh, Koll.

*Drafting of the manuscript*: Wenzel, Humke, Mandel.

*Critical revision of the manuscript for important intellectual content*: Tilki, Steuber, Graefen, Banek, Kluth, Chun.

*Statistical analysis*: Wenzel, Siech, Mandel.

*Obtaining funding*: None.

*Administrative, technical, or material support*: None.

*Supervision*: Tilki, Steuber, Graefen, Banek, Kluth, Chun.

*Other*: None.

  ***Financial disclosures:*** Mike Wenzel certifies that all conflicts of interest, including specific financial interests and relationships and affiliations relevant to the subject matter or materials discussed in the manuscript (eg, employment/affiliation, grants or funding, consultancies, honoraria, stock ownership or options, expert testimony, royalties, or patents filed, received, or pending), are the following: None.

  ***Funding/Support and role of the sponsor:*** None.

  ***Acknowledgments:*** This study was part of the EPIC-REAP (Enhancing Prostate cancer care In Germany Combining Real-world data And AI for Enhanced Analysis and Precision) project supported by the Mildred-Scheel Nachwuchszentrum Frankfurt.

  ***Data sharing:*** Data are available for bona fide researchers who request it from the authors.
